# Anthropometric trends and the risk of cardiovascular disease mortality in a Lithuanian urban population aged 45–64 years

**DOI:** 10.1177/1403494815597582

**Published:** 2015-12

**Authors:** Dalia Luksiene, Abdonas Tamosiunas, Dalia Virviciute, Gailute Bernotiene, Anne Peasey

**Affiliations:** 1 Lithuanian University of Health Sciences, Academy of Medicine, Institute of Cardiology, Department of Population Studies, Lithuania; 2 Lithuanian University of Health Sciences, Academy of Medicine, Public Health Faculty, Department of Environmental and Occupational Medicine, Lithuania; 3 Lithuanian University of Health Sciences, Academy of Medicine, Public Health Faculty, Department of Preventive Medicine, Lithuania; 4 Department of Epidemiology and Public Health, University College London, UK

**Keywords:** body mass index, waist circumference, waist:hip ratio, waist:height ratio, cardiovascular disease

## Abstract

*Aims:* To estimate trends in anthropometric indexes from 1992 to 2008 and to evaluate the risk of cardiovascular disease mortality in relation to anthropometric indexes (body mass index, waist circumference, waist:hip ratio, waist:height ratio). *Methods:* Data from the three surveys (1992–2008) are presented. A random sample of 5147 subjects aged 45–64 years was selected for statistical analysis. During follow-up there were 141 deaths from cardiovascular disease (excluding those with cardiovascular disease at entry). Cox’s regression was used to estimate the associations between anthropometric indexes and cardiovascular disease mortality. *Results:* During a 17-year period among men, the prevalence of obesity (body mass index ⩾30 kg/m^2^) increased from 18.4% to 32.1% (*p*<0.001) and a high level of waist:hip ratio (>0.9) from 59.3% to 72.9% (*p*<0.001). The risk profile of obesity did not change in women, but prevalence of a high level of waist:hip ratio (>0.85) increased from 25.9% to 41.5% (*p*<0.001). Multivariable-adjusted Cox’s regression models showed that body mass index, waist circumference, waist:hip ratio, waist:height ratio were associated with cardiovascular disease mortality risk only in men (hazard ratios 1.40, 1.45, 1.49, 1.46 respectively (*p*<0.01)). ***Conclusions:* Our data indicate that anthropometric measures such as body mass index, waist circumference, waist:hip ratio and waist:height ratio are good indicators of cardiovascular disease mortality risk only in men aged 45–64 years.**

## Introduction

Data on cardiovascular disease (CVD) mortality in relation to various anthropometric measures of obesity are still inconsistent. In recent years, discussion has increased about which measure of overweight and obesity is the best indicator to identify those individuals who are at high CVD risk [1,2]. The most popular measures are height and weight, expressed preferably as body mass index (BMI). However, recent research shows that BMI cannot make the distinction between an elevated body weight due to high levels of lean compared to fat body mass and an excess of body fat is more frequently associated with metabolic abnormalities than a high level of lean body mass [1]. The measurement of the waist circumference (WC) is used mostly for defining central obesity, but this measurement has been criticized for not taking into account differences in body height [3]. It has been proven that an individual’s percentage of body fat is higher in shorter people compared to taller people with an equivalent BMI [4]. Recent studies have found that waist:height ratio (WHtR) may be used as marker of body fat centralization, which could be an efficient predictor of CVD risk [3,5]. Measures of WC, waist:hip ratio (WHR) and WHtR, which more accurately describe the distribution of body fat compared with BMI, have been suggested to be more closely associated with clinical outcomes and mortality [2,6].

Some investigators show that the strength of the association between anthropometric indexes and cardiovascular risk is population-dependent [7]. Different anthropometric indexes of regional adiposity have been proposed for identifying persons at higher risk of death; however, research studies specifically assessing these indexes in large cohorts are insufficient [8]. The aim of this study was to estimate the trends in anthropometric indexes from 1992 to 2008 and to evaluate the risk of CVD mortality in relation to anthropometric indexes.

## Materials and methods

### Study sample

Data from the three surveys are presented in this article. The first survey in the framework of the Multinational Monitoring of Trends and Determinants in Cardiovascular Disease (MONICA) study was performed in 1992–1993. The second survey was conducted in 2001–2002 in accordance with MONICA study protocol. The third survey was performed in 2006–2008 in the framework of the Health, Alcohol and Psychosocial Factors in Eastern Europe (HAPIEE) study [9]. These surveys were carried out in Kaunas city with a population size of 348,624. All three random samples of men and women aged 45–64 years, stratified by gender and age, were randomly selected from the Kaunas population register data. The response rates were: for the first survey − 58.6%; for the second − 62.4%; for the third − 58.1%. Data from 5147 subjects who were examined for all anthropometric measurements and analysed CVD risk factors were approved for statistical analysis. Data of 200 subjects were excluded from the statistical analysis because of incomplete information about the anthropometric measurements and/or analysed covariates.

### Ethics statement

All three studies were approved by the Regional Biomedical Research Ethics Committee at the Lithuanian University of Health Sciences and the HAPIEE study (2006–2008) and also by the UCLH Research Ethics Committee Alpha at University College London, UK. All respondents provided written informed consent.

### Baseline interview

A baseline interview was carried out in each survey. Blood pressure (BP), weight and height measurements and laboratory analyses were conducted following the same methodology and the same or comparable questionnaires were used.

### Anthropometric and clinical measurements

Weight and height were measured with a calibrated medical scale and without shoes or heavy clothes. BMI was calculated as the weight in kilograms divided by the height in metres squared (kg/m^2^). Normal weight was defined as BMI 18.5–24.99 kg/m², overweight as BMI 25.0–29.99 kg/m² and obesity as BMI ⩾30.0 kg/m². Insufficient weight was defined as BMI <18.5 kg/m².

Waist and hip circumferences were measured (without upper clothes) by standard tape with an accuracy of 0.5 cm. The diagnostic criteria for elevated WC by the American Heart Association, National Heart, Lung, and Blood Institute Scientific Statement recommendations are for men WC ⩾102 cm and for women ⩾88 cm [10]. The criterion of abdominal obesity was defined as WC in centimetres divided by the hip circumference in centimetres, thus for men WHR >0.9 and for women WHR >0.85 was defined as abdominal obesity [10]. WHtR was calculated as WC divided by height.

BP was measured twice using a mercury sphygmomanometer and appropriately sized arm cuffs on the right arm. The initial measurement was performed after 5 minutes rest. After 2 minutes, the second measurement was made. The Korotkoff phase 1 (beginning of the sound) and the fifth phase of Korotkoff (disappearance of the sound) was recorded as systolic BP (SBP) and diastolic BP (DPB) respectively. The mean of the two readings was used. Arterial hypertension (AH) was defined as mean SBP ⩾140 mmHg and/or mean DBP ⩾90 mmHg or respondent reported taking medicine for high BP in the last 2 weeks.

In all surveys, CVD was determined according to following criteria. Coronary heart disease (CHD) was determined by: (1) a documented history of myocardial infarction (MI) and/or ischaemic changes on an electrocardiogram (ECG) coded by the Minnesota codes (MC) 1-1 or 1-2 [11]; (2) angina pectoris was defined by G. Rose’s questionnaire (without MI and/or MC 1-1 or 1-2; (3) [12]; ECG findings by MC 1-3, 4-1, 4-2, 4-3, 5-1, 5-2, 5-3, 6-1, 6-2, 7-1, 8-3 (without MI and/or MC 1-1, 1-2 and without angina pectoris). Stroke was determined using the question: ‘Has a doctor ever told you that you have had a stroke?’

### Laboratory analyses

Fasting blood samples were taken. Serum samples from the first two surveys were analysed in the Laboratory of the Institute of Cardiology of the Lithuanian University of Health Sciences and serum samples from the third survey in the WHO Regional Lipid Reference Centre, Institute of Clinical and Experimental Medicine, Prague (Czech Republic). Serum lipid concentrations were measured, using a conventional enzymatic method. An elevated triglycerides level was defined as ⩾1.70 mmol/L [10]. Concentration of glucose in capillary blood was determined by an individual glucometer ‘Glucotrend’ [13]. A high glucose level was defined as fasting glucose level ⩾6.10 mmol/L.

### Covariates

The respondent’s age, education, smoking status and physical activity were collected using a standard questionnaire. Education was classified into four education levels: primary; incomplete secondary; secondary or college; university.

Smoking habits were classified to three groups: current smokers; former smokers; never smokers. A subject who smoked at least one cigarette per day was classified as a current smoker. Physical activity was assessed by asking about physically demanding activities in a typical week in summer and winter seasons, active transportation to work and physically demanding activities, such as housework, gardening and maintenance of the house, in addition to engagement in sports, games or hiking. Subjects who were physically active less than 10 hours per week were classified as physically inactive.

### Follow-up

The participants were followed up from the beginning of each baseline health examination until 31 December 2013 and mortality data were extracted from the regional mortality register. The outcome measure of interest was CVD mortality (excluding those with a documented history of CVD at entry). CVD mortality included CHD, stroke and other vascular causes and was defined as 390–458 – codes of 9th ICD and I00–199 – codes of 10th ICD). There were 141 deaths from CVD (excluding those with previous CVD at entry) (96 men and 45 women). The mean duration and SD of follow-up was 8.98±5.12 years among men and 9.49±5.03 years among women.

### Statistical analysis

All the analyses were performed separately for men and women. Descriptive statistics (prevalence rates, means and SD) were calculated for variables in each survey. Linear-by-linear association test (test for linearity) to assess time trends from 1992 to 2008 was performed and *p*<0.05 was defined as statistically significant. All surveys were weight adjusted with age to the Kaunas population census of 2006. In 2006, 55% (57.0% of men and 53.5% of women) were aged 45–54 years and 45% (43.0% of men and 46.5 of women) were aged 55–64 years. The weights were obtained by dividing the population percentage by the surveys percentage. Pearson’s correlation coefficients between anthropometric indexes and CVD risk factors were calculated.

The association between anthropometric indexes (BMI, WC, WHR, WHtR) and risk of mortality from CVD mortality (excluding those with previous CVD at entry) was investigated using Cox’s proportional hazards regression analysis. First, the association between anthropometry indexes and mortality changes between surveys was evaluated using likelihood ratio test and the results were not statistically significant (*p*>0.05). Thus, we calculated the association between anthropometric indexes and risk of mortality from CVD over all three surveys, putting survey year in a Cox’s proportional hazards regression model as a strata variable. Hazard ratios (HRs) represented a change in mortality per 1 unit of the anthropometric index and per 1 SD change in anthropometric indexes. HR was adjusted by age (Model 1) and by age, education, AH, fasting glucose and triglyceride levels, smoking and physical activity (Model 2). All analyses were carried out using SPSS version 13 and STATA IC version 10.

## Results

Baseline characteristics according to anthropometric indexes of three cohorts aged 45–64 years are presented in Table I. During the period 1992–2008 generally negative changes in anthropometric indexes profile were observed in men: the mean levels of BMI, WC, WHR and WHtR increased significantly (*p*<0.001). The prevalence of normal weight and overweight decreased, while the prevalence of obesity increased over this period (*p*<0.05). Also during this period, the prevalence of high levels of WC and high levels of WHR increased. The risk profile of overweight and obesity did not change in women, but the prevalence of high levels of WHR and the mean levels of WC, WHR and WHtR increased.

**Table I.** Characteristics of subjects aged 45–64 years in three cohorts (data adjusted by age).

Characteristics	1992–1993	2001–2002	2006–2008	*P* for trend
Men	(*N*=389)	(*N*=420)	(*N*=1528)	
Mean age, years (SD)	52.9 (5.69)	52.9 (5.42)	54.9 (5.69)	<0.001
Weight, kg, mean (SD)	82.8 (12.8)	84.5 (14.5)	87.2 (15.9)	<0.001
Height, cm, mean (SD)	174.5 (6.41)	174.3 (6.19)	175.1 (6.41)	0.021
BMI, kg/m², mean (SD)	27.2 (3.91)	27.7 (4.23)	28.4 (4.71)	<0.001
BMI, kg/m², %, (95% CI)				
<18.5	0	0.2 (–0.2 to 0.6)	0.2 (0–0.4)	0.434
18.5–24.99	31.7 (27.1–36.3)	26.9 (22.6–31.0)	23.7 (21.7–25.9)	0.001
25.0–29.9	49.9 (44.9–54.9)	45.5 (40.6–50.2)	44.0 (41.5–46.5)	0.039
⩾30.0	18.4 (14.5–22.1)	27.4 (23.1–31.7)	32.1 (29.8–34.4)	<0.001
WC, cm, mean (SD)	92.4 (10.1)	93.1 (10.9)	95.1 (12.5)	<0.001
WC ⩾102 cm, %, (95% CI)	17.1 (13.4–20.8)	19.5 (15.7–23.3)	26.5 (24.3–28.7)	<0.001
WHR, mean (SD)	0.92 (0.06)	0.93 (0.07)	0.94 (0.07)	<0.001
WHR >0.9, %, (95% CI)	59.3 (54.4–64.2)	68.4 (64.0–72.8)	72.9 (70.7–75.1)	<0.001
WHtR, mean (SD)	0.53 (0.06)	0.53 (0.06)	0.54 (0.07)	<0.001
Mortality from CVD^**a**^				
Number of events	52	17	27	
Person years	5,818.7	3,771.1	8,205.2	
Follow-up years, mean (SD)	18.5 (5.6)	11.3 (2.5)	6.4 (1.1)	
Mortality/10000 person years	87.8 (63.8–112)	43.7 (22.6–64.7)	26.09 (15.1–37.1)	
Women	(*N*=401)	(*N*=551)	(*N*=1858)	
Mean age, years (SD)	53.3 (5.42)	52.9 (5.39)	55.3 (5.70)	<0.001
Weight, kg, mean (SD)	75.3 (13.5)	77.1 (15.9)	76.4 (15.2)	0.421
Height, cm, mean (SD)	161.8 (5.27)	161.2 (6.07)	161.7 (5.92)	0.642
BMI, kg/m², mean (SD)	28.8 (5.15)	29.6 (5.76)	29.3 (5.79)	0.422
BMI, kg/m², %, (95% CI)				
<18.5	0.2 (–0.2 to 0.6)	0.3 (–0.1 to 0.8)	0.2 (0–0.4)	0.816
18.5–24.99	27.9 (22.8–31.6)	22.2 (18.7–25.7)	24.9 (22.9–26.9)	0.681
25.0–29.9	35.0 (30.2–39.6)	32.6 (28.7–36.5)	36.5 (34.3–38.7)	0.254
⩾30.0	36.9 (31.6–41.0)	44.9 (40.6–49.0)	38.4 (36.2–40.6)	0.808
WC, cm; mean (SD)	86.1 (11.8)	86.7 (12.9)	87.8 (13.7)	0.009
WC ⩾88 cm, %, (95% CI)	43.4 (38.5–48.3)	46.3 (42.1–50.5)	45.6 (43.3–47.9)	0.558
WHR, mean (SD)	0.81 (0.07)	0.82 (0.08)	0.84 (0.07)	<0.001
WHR >0.85, %, (95% CI)	25.9 (21.6–30.2)	34.1 (30.1–38.1)	41.5 (39.3–43.7)	<0.001
WHtR, mean (SD)	0.53 (0.08)	0.54 (0.08)	0.54 (0.09)	0.011
Mortality from CVD^**a**^				
Number of events	32	7	6	
Follow-up years, mean (SD)	20.3 (3.1)	12.2 (1.1)	6.6 (0.8)	
Mortality/10000 person years	53.9 (36.2–71.6)	16.5 (5.5–27.5)	5.02 (0.6–9.4)	

CVD: cardiovascular disease; CI: confidence interval; BMI: body mass index; WC: waist circumference; WHR: waist:hip ratio; WHtR: waist:height ratio.

a Mortality from CVD excluded those with previous CVD at entry.

Correlation coefficients among anthropometric measures in a Kaunas urban population aged 45–64 years (Table II) indicate that BMI, WC, WHR and WHtR were strongly correlated in men and women, suggesting that measures of obesity based on these parameters will provide comparable information. However, WHR showed a weaker correlation with the other anthropometric measurements in both groups. The anthropometric indexes were significantly associated with most CVD risk factors (covariates) in men and women groups (see table in supplementary material).

**Table II.** Pearson’s correlation coefficient among anthropometric measures in a Kaunas urban population aged 45–64 years (data adjusted by age).

	Men 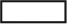 (*N*=2337)		Women 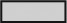 (*N*=2810)	
	BMI	WC	WHR	WHtR
BMI	–	0.908	0.531	0.915
WC	0.917	–	0.761	0.972
WHR	0.690	0.834	–	0.762
WHtR	0.923	0.957	0.842	–

BMI: body mass index; WC: waist circumference; WHR: waist:hip ratio; WHtR: waist:height ratio. All correlation coefficients are significant (*p*<0.01).

Characteristics of respondents according to vital status and cause of death are shown in Table III. Men and women who died from CVD were older, lower educated, more physically inactive and had a higher AH rate (only in women) than those alive at the end of follow up, although BMI, WC, WHR and WHtR did not differ. Meanwhile, in the women group, those who died from CVD had a higher WC, WHR and WHtR than those alive at the end of follow-up.

**Table III.** Characteristics of the subjects aged 45–64 years according to vital status and cause of death.

Characteristics	Men	Alive	Women	Alive
	CVD deaths^a^	*N*=1962	CVD deaths^a^	*N*=2637
	*N*=96		*N*=45	
Mean age, years (SD)	55.8 (5.90)^**c**^	53.9 (5.63)	58.9 (4.22)^**d**^	54.3 (5.67)
BMI, kg/m², mean (SD)	28.3 (5.49)	28.1 (4.43)	30.6 (5.40)	29.2 (5.70)
WC, cm; mean (SD)	96.0 (13.4)	94.2 (11.8)	90.8 (12.4)^**c**^	87.0 (13.2)
WHR, mean (SD)	0.95 (0.07)	0.94 (0.07)	0.86 (0.08)^**d**^	0.83 (0.07)
WHtR, mean (SD)	0.55 (0.08)	0.54 (0.07)	0.57 (0.08)^**b**^	0.54 (0.08)
Tg, mean (SD)	1.46 (0.77)	1.56 (1.08)	1.41 (0.90)	1.38 (0.79)
FG, mean (SD)	5.95 (2.52)	5.64 (1.11)	6.39 (3.79)	5.65 (1.14)
AH, %	76.7	69.9	81.6^**c**^	59.5
Smokers, %				
Former	26.7	27.0	5.1	7.8
Current	44.2	37.4	5.1	13.0
Education lower than secondary, %	38.4^**c**^	25.1	47.4^**b**^	28.4
Inactive leisure physical activity, %	43.7^**b**^	33.1	52.6^**d**^	24.3

CVD: cardiovascular disease; BMI: body mass index; WC: waist circumference; WHR: waist:hip ratio; WHtR: waist:height ratio; SBP: systolic blood pressure; DBP: diastolic blood pressure; AH: arterial hypertension (SBP ⩾140 and/or DBP ⩾90 mm Hg or treatment); Tg: triglycerides; FG: fasting glucose.

Data adjusted by age and stratified by study survey year.

a CVD deaths excluded those with previous CVD at the entry.

b *p*<0.05.

c *p*<0.01.

d *p*<0.001 compared with alive group.

We evaluated the risk of CVD mortality in relation to presence of anthropometric indexes. First, we analysed CVD mortality risk per 1 unit increase of each the anthropometric index (data adjusted by age, education, AH, fasting glucose level, triglycerides level, smoking and physical activity habits). The results show that only among men was an increase of BMI by 1 kg/m² and WC by 1 cm associated with a higher risk of CVD death by 8% (HR=1.08, 95% confidence interval (CI) 1.03–1.13, *p*=0.003) and by 3% (HR=1.03, 95% CI 1.01–1.05, *p*=0.002) respectively.

CVD mortality risk per 1 SD increase in anthropometric indexes in a population aged 45–64 years is shown in Table IV. After adjustment for age (Model 1), all four anthropometrics measures (BMI, WC, WHR, WHtR) were associated with CVD mortality risk in the men and women groups (except for BMI and WC in women). After additional adjustment (Model 2), the four anthropometric measures were associated with CVD mortality risk only in men (from 40% to 49%). However, among women after additional adjustment (Model 2), all four anthropometric measures were not associated with CVD mortality risk.

**Table IV.** CVD mortality risk^**a**^ per 1 SD^b^ increase in anthropometric indexes in subjects aged 45–64 years (Kaunas urban population linked mortality file).

Anthropometric indexes	Men		Women	
	HR (95% CI)	HR (95% CI)	HR (95% CI)	HR (95% CI)
	Model 1^c^	Model 2^d^	Model 1^c^	Model 2^d^
BMI, kg/m²	**1.32 (1.08–1.62)**	**1.40 (1.12–1.75)**	1.26 (0.97–1.63)	1.06 (0.79–1.44)
WC, cm	**1.41 (1.15–1.73)**	**1.45 (1.15–1.82)**	1.36 (0.99–1.85)	1.08 (0.75–1.53)
WHR	**1.54 (1.25–1.89)**	**1.49 (1.19–1.87)**	**1.66 (1.18–2.35)**	1.32 (0.91–1.92)
WHtR	**1.43 (1.18–1.74)**	**1.46 (1.17–1.82)**	**1.37 (1.01–1.86)**	1.08 (0.75–1.54)

HR: hazard ratio; CI: confidence interval; BMI: body mass index; WC: waist circumference; WHR: waist:hip ratio; WHtR: waist:height ratio; CVD: cardiovascular disease.

*Note*. Some values in Table IV are shown in bold because the HR indicates statistically significant change in CVD risk per 1 SD increase of the anthropometric indexes.

a The risk of mortality from CVD was calculated excluding those responders with previous CVD at entry.

b 1 SD for anthropometric indexes in the men group: BMI (4.55 kg/m^2^ ); WC (12.0 cm); WHR (0.071); WHtR (0.068); 1 SD for anthropometric indexes in the women group: BMI (5.69 kg/m^2^); WC (13.3 cm); WHR (0.074); WHtR (0.085).

c HR adjusted by age and data stratified by study survey year.

d HR adjusted by age, education, blood pressure, fasting glucose level, triglycerides level, smoking, physical activity habits, and data stratified by study survey year.

## Discussion

Obesity is a major risk factor for development of chronic diseases and an important cause of mortality [2,5,8]. The Lithuanian population is characterized by high morbidity and mortality from CVD and a high prevalence of obesity [14,15]. Our study provides the first population-based information on trends of the prevalence of overweight, obesity, increased WC and WHR over a 17-year period (1992–2008) in Lithuania. The findings in our longitudinal study show significant changes. The prevalence of obesity in men increased over this 17-year period from 18.4% to 32.1% and the prevalence of high levels of WHR (>0.9) increased from 59.3% to 72.9%. In women, the risk profile of obesity did not change; however, the prevalence of high levels of WHR (>0.85) increased from 25.9% to 41.5%. This is comparable to the USA, where in 1988–1994 to 1999–2000 in NHANES surveys, the prevalence of obesity in adults in the USA increased from 23% to 31% [16,17]. Currently, the USA has reportedly the highest prevalence of obesity in the adult population (31%), which is nearly 10 times higher than Japan (3.8% in men and 3.2% in women) [17,18]. The data from the Stockholm County 1990-2010 Study show that during the period 1990–2010, the prevalence of obesity increased from 5.5% to 11.2% in men and from 4.6% to 10.3% in women [19]. Our data add to this body of evidence, indicating that the prevalence of obesity has increased over the past 20 years. Body composition is currently recognized as one of the most important global challenges for public health.

The recent gender-specific research analyses how diseases differ between men and women, for example, risk factors for CVD have a different impact in men and women [20]. According to new research data, there are some differences between reasons for obesity among men and women. In women, BMI is associated with increased body shame, dietary restraint and depressive symptoms, while in men BMI is associated with concerns about shape and weight but not dietary restraint [21].

Persistent obesity dysregulates metabolic processes, including action of insulin on glucose lipid-free fatty acid metabolism, and severely affects processes controlling blood glucose levels, BP and lipids [22]. This leads to the development of metabolic syndrome, which comprises a cluster of conditions including hyperglycaemia, dyslipidaemia and AH. Many research studies indicate that BMI, WC, WHR and WHtR are significantly correlated with CVD risk factors, such as dyslipidaemia, AH, type 2 diabetes [2,23,24]. The findings in our study show that all analysed anthropometric indexes are significantly associated with CVD risk factors; for this reason, those covariates were included in a multivariable regression analysis.

As well as examining time trends in anthropometric indexes, our study evaluated associations between anthropometric indexes and risk of CVD mortality. The results show that, among men, a 1 unit increase in BMI and WC were associated with an increased risk of CVD death by 8% and 3% respectively (*p*=0.003). One SD increases in all four anthropometric measures in men were associated with increased CVD mortality risk. Data from the PAMELA study show, that a 1 unit increase in baseline BMI and WC were associated with an increased risk of CVD death by 12% (for baseline BMI) and 5% (for baseline WC), respectively. After adjustment for confounders, only the increased risk of CVD death associated with higher baseline BMI remained significant [25]. Data from European DECODE Study of over 46,000 men and women aged 24–99 years (median follow-up of 7.9 years) showed that all four anthropometric measures of abdominal obesity (BMI, WC, WHtR, WHR) had positive associations with CVD mortality [26]. The PREDIMED Study also assessed the associations between all four anthropometric measurements (WC, WHtR, BMI and height) and mortality (with 4.8 years of follow-up), but BMI exhibited weaker associations with mortality than WC or WHtR [8].

Furthermore, recent studies have suggested that BMI should be refined by measuring additional indexes of fat distribution, namely WC, WHR or WHtR, to better identify higher-risk subjects [1,27]. WC and WHtR are reportedly better anthropometric measures of abdominal obesity than BMI and have a stronger correlation with intra-abdominal fat content and cardiometabolic risk factors [6,26,28]. Some studies show that WHtR is a better predictor of risk factors for CVD and risk of CVD mortality than WC [3,6,29]. The findings in our longitudinal study show that all analysed anthropometric indexes significantly positively correlated with CVD risk factors for both men and women. However, multivariate Cox’s regressions demonstrated that significant associations with CVD mortality risk was found only in men. In women, the main roles played were AH and physical inactivity. Therefore, after additional including of AH, physical inactivity and other covariates, the CVD risk estimates for anthropometric measures among women decreased.

### Limitation

It is important to be aware of several limitations of our results. First, the present study did not examine a national sample, but rather included only a random sample of 45–64 year olds of an urban population of one city (Kaunas is the second largest city in Lithuania); however, our response rates are comparable with other longitudinal studies. Further study is needed to examine these associations in other ages or in the rural population. Second, healthy nutritional habits are an important determinant of obesity. Diet status evaluation in these cohorts was based on a food frequency questionnaire, but only a few questions were comparable across all three surveys. For this reason, we did not include a measure of nutrition habits in the statistical analyses.

The strength of our study includes its large and prospective nature, which makes selection and information bias less likely. Numerator-denominator bias is minimized through linkage of the survey cohorts with the mortality register, rather than relying on direct contact with participants or their relatives. A common protocol also reduces the likelihood of bias. In addition, with a wealth of phenotypic data collected, we have been able to adjust for a range of potential confounding variables, including age, education, AH, fasting glucose level, triglycerides level, smoking and physical activity habits in the analyses that are presented.

## Conclusion

The results of this prospective study suggest that over a 17-year period the prevalence of obesity and high level of WHR has increased in men. The risk profile of obesity did not change in women, but the prevalence of high levels of WHR increased. A significant increase in the risk for CVD mortality was associated with increased BMI, WC, WHR and WHtR in men.

## Supplementary Material

Supplementary material
