# Supplementary material for: Anthropometric trends and the risk of cardiovascular disease mortality in a Lithuanian urban population aged 45–64 years
Source: Scand J Public Health. 2015 Dec;43(8):882–9. doi: 10.1177/1403494815597582 (PMC4639754; doi:10.1177/1403494815597582)
Supplement: Supplementary material [file Supplementary_Table.pdf]

Table S I. The association between anthropometric indexes and cardiovascular risk factors

Covariates		BMI		WC		WHR		WHtR	
	N	Mean (SD)	p	Mean (SD)	p	Mean (SD)	p	Mean (SD)	p
**MEN**									
**Triglycerides**									
<1.7 mmol/L	1614	27.2 (4.16)	<0.001	91.8 (11.1)	<0.001	0.92 (0.07)	<0.001	0.53 (0.06)	<0.001
≥1.7 mmol/L	723	29.9 (4.67)		99.6 (11.9)		0.97 (0.07)		0.57 (0.07)	
**Fasting glucose**									
<6.10 mmol/L	1692	27.2 (4.01)	<0.001	92.0 (10.8)	<0.001	0.93 (0.07)	<0.001	0.53 (0.06)	<0.001
≥6.10 mmol/L	645	30.2 (5.03)		99.9 (12.8)		0.96 (0.07)		0.57 (0.07)	
**AH**									
No	713	26.1 (3.7)	<0.001	88.9 (10.0)	<0.001	0.90 (0.06)	<0.001	0.51 (0.06)	<0.001
Yes	1616	28.9 (4.6)		96.6 (11.9)		0.95 (0.07)		0.55 (0.07)	
**Smoking status**									
Never smokers	796	28.1 (4.09)	<0.001	93.7 (11.1)	<0.001	0.93 (0.07)	<0.001	0.54 (0.06)	<0.001
Former	631	29.3 (4.58)		97.6 (12.0)		0.95 (0.07)		0.56 (0.07)	
Current	910	27.2 (4.62)		92.3 (11.9)		0.93 (0.07)		0.53 (0.07)	
**Education**									
Secondary &higher	1715	27.9 (4.44)	0.250	93.9 (11.8)	0.080	0.93 (0.07)	0.001	0.54 (0.07)	0.013
Lower than secondary	622	28.2 (4.70)		94.9 (12.2)		0.94 (0.07)		0.55 (0.07)	
**Physical activity**									
Active	1520	28.0 (4.50)	0.549	93.9 (11.7)	0.064	0.93 (0.07)	0.003	0.54 (0.07)	0.159
Inactive	817	28.1 (4.54)		94.8 (12.3)		0.94 (0.07)		0.54 (0.07)	
**WOMEN**									
**Triglycerides**									
<1.7 mmol/L	2132	28.4 (5.36)	<0.001	84.9 (12.5)	<0.001	0.82 (0.07)	<0.001	0.53 (0.08)	<0.001
≥1.7 mmol/L	678	32.0 (5.85)		94.7 (13.1)		0.87 (0.07)		0.59 (0.08)	
**Fasting glucose**									
<6.10 mmol/L	2090	28.5 (5.39)	<0.001	85.1 (12.5)	<0.001	0.82 (0.07)	<0.001	0.53 (0.08)	<0.001
≥6.10 mmol/L	720	31.6 (5.90)		93.6 (13.6)		0.86 (0.07)		0.58 (0.09)	
**AH**									
No	1116	26.9 (4.7)	<0.001	81.6 (11.0)	<0.001	0.80 (0.07)	<0.001	0.50 (0.07)	<0.001
Yes	1692	30.8(5.8)		91.0 (13.4)		0.85 (0.07)		0.57 (0.08)	
**Smoking status**									
Never smokers	2237	29.5 (5.69)	<0.001	87.6 (13.3)	0.033	0.83 (0.07)	0.042	0.54 (0.09)	0.003
Former	215	28.9 (5.92)		87.5 (13.3)		0.83 (0.07)		0.54 (0.08)	
Current	358	27.9 (5.36)		85.6 (13.4)		0.84 (0.08)		0.53 (0.08)	
**Education**									

Secondary &higher	1992	28.9 (5.65)	<0.001	86.4 (13.3)	<0.001	0.82 (0.08)	<0.001	0.53 (0.08)	<0.001
Lower than secondary	818	29.9 (5.75)		89.4 (13.1)		0.84 (0.07)		0.56 (0.08)	
**Physical activity**									
Active	2099	29.1 (5.53)	0.039	86.9 (13.0)	0.027	0.83 (0.07)	0.091	0.54 (0.08)	0.025
Inactive	711	29.7 (6.15)		88.3 (13.9)		0.83 (0.08)		0.55 (0.09)	

N: number of cases; SD: standard deviation; BMI: body mass index; WC: Waist circumference; WHR: Waist-hip-ratio; WHtR: Waist-to-height ratio; AH: arterial hypertension (SBP≥140 and/or DBP≥90 mm Hg or treatment)
